# Latin America and the Caribbean: Socioeconomic Realities and Implications for Health and HIV Responses

**DOI:** 10.1002/jia2.70174

**Published:** 2026-07-25

**Authors:** Claudia P. Cortes, Carmen Pérez Casas, Pedro Cahn

**Affiliations:** ^1^ Centre for HIV/AIDS Integral Research, Faculty of Medicine Universidad de Chile Santiago Chile; ^2^ Strategy Lead, HIV and Pandemics Unitaid Geneve Switzerland; ^3^ Fundación Huésped Buenos Aires Argentina

Latin America and the Caribbean (LAC) is a region of remarkable diversity, encompassing more than 650 million people across countries and territories with different levels of economic development, political stability, health‐system capacity and social cohesion. This diversity is one of the region's strengths. Several countries have built robust public health infrastructures, expanded access to antiretroviral therapy (ART), promoted human‐rights‐based approaches to HIV and supported meaningful community participation in health policymaking. These achievements, however, coexist with profound and persistent inequities.

Although almost 70% of countries in the region are currently classified by the World Bank as middle‐income economies, this classification often obscures more than it clarifies [1]. Income levels illustrate this heterogeneity: in 2024, Atlas‐method gross domestic product per capita ranged from approximately US$2142 in Haiti (living on less than US$3.00/day) to more than US$23,900 in Uruguay, while several Caribbean economies were classified as high‐income [1, 2]. Yet, national averages can conceal deep within‐country inequalities. LAC remains one of the most unequal regions in the world, with wealth, opportunities and access to services concentrated among relatively small segments of the population [3]. Regional economic growth in the first decade of the twenty‐first century was real, but its gains were uneven and fragile. In addition, while poverty declined before the COVID‐19 pandemic, subsequent economic disruption, inflationary pressures, high levels of economic and employment informality and growing public debt have limited the capacity of governments to expand social protection and strengthen health systems.

Urbanization and migration further shape regional HIV responses. More than 80% of the population lives in urban settings, making LAC one of the most urbanized regions globally. Cities can improve access to education and health services, but they also concentrate poverty, overcrowding, violence and exclusion. Informal settlements and marginalized urban communities often face barriers to HIV prevention, testing and treatment. At the same time, migration has required health systems to adapt, as these mobile populations present with documentation barriers, interrupted care pathways and changing patterns of vulnerability.

These structural realities intersect with persistent discrimination affecting gay men and other men who have sex with men, transgender people, sex workers, people who use drugs, migrants, Indigenous communities, people of African descent and people living in poverty. These inequities are visible in the epidemic itself: in Latin America, estimated adult HIV prevalence is 0.6%, yet median prevalence among reporting countries is 8.2% among gay men and other men who have sex with men and 9.5% among transgender people; high prevalence has also been reported among non‐migrating Afro‐descendant and Indigenous populations, including in Brazil, where HIV prevalence among Afro‐descendant women has been reported to be twice that of the overall female population [4]. Gender‐based violence, stigma, criminalization and limited economic opportunities continue to facilitate HIV acquisition and undermine retention in care. Coinfections, including tuberculosis, viral hepatitis, sexually transmitted infections and endemic or neglected diseases, add further complexity to HIV care in many LAC settings. Recent political changes in several countries, particularly the ascent of far‐right parties to government, have exacerbated the situation by targeting key populations and imposing budgetary restrictions that undermine essential services and protections.

The global HIV response has also entered a period of acute financial and political uncertainty. Although LAC is not highly dependent overall on external assistance for HIV treatment and domestic funding supports most ART programmes, recent reductions in development financing have exposed the vulnerability of prevention services, support for key populations and migrants, and community‐led responses [5]. For a region in which many countries are considered too wealthy for donor support and procurement of HIV commodities but still lack sufficient capacity to fund HIV programmes in their entirety, the middle‐income label can become a barrier to equitable access.

This special issue of the *Journal of the International AIDS Society* focuses on this tension within a region with substantial scientific, clinical and community capacity, but where structural inequalities and uneven access shape HIV outcomes. Recent UNAIDS estimates for 2025 indicate that approximately 2.84 million people were living with HIV in LAC, including 2.5 million in Latin America and 340,000 in the Caribbean. Latin America recorded a 13% increase in new HIV acquisitions between 2010 and 2024, from 110,000 to 120,000 annually, while the Caribbean recorded a 21% decline, from 19,000 to 15,000 annually [4, 6]. AIDS‐related deaths declined by 31% in Latin America and by 62% in the Caribbean over the same period, yet late diagnosis, treatment interruptions and incomplete ART coverage remain central concerns [4, 6]. Viral suppression, estimated at 66% of people living with HIV in both subregions, remains below the level required to achieve the 95‐95‐95 targets [4, 6, 7]. These trends reflect inequities in access to prevention, diagnosis, treatment, monitoring, social protection and rights‐based care.

The contributions in this issue illuminate these tensions from complementary perspectives. Prochazka and colleagues [8] provide a broad regional framing, situating the epidemic within the demographic, epidemiological and policy landscape of the region and emphasizing heterogeneity and the need for strategies grounded in local realities rather than imported assumptions.

ten Brink and colleagues [9] bring a health economics and modelling perspective to one of the most urgent questions now facing the region: how to maximize impact in constrained financing environments. Their work is especially relevant as countries are forced to make difficult decisions about prevention priorities, resource allocation and sustainability. Cifuentes and colleagues [10] extend this discussion to the political economy of access. Their viewpoint reminds us that equitable HIV care depends not only on clinical guidelines, but also on intellectual property, procurement, political will and regional capacity to negotiate access to essential medicines in a regional pharmaceutical sovereignty.

Access to innovation is a particularly important test case for LAC. Long‐acting agents have the potential to transform HIV prevention and treatment, especially for people for whom daily oral medication is difficult because of stigma, mobility, violence, confidentiality concerns or unstable access to services. However, these benefits will matter only if the tools are affordable and implemented equitably. LAC has contributed to pivotal evidence for long‐acting prevention, yet countries in the region remain at risk of delayed or uneven implementation. The challenge is not only whether these tools work, but whether the populations and health systems that helped generate the evidence will be able to benefit from it.

Two contributions in this issue address prevention implementation in key populations. Garcia and colleagues [11] provide real‐world evidence from Buenos Aires, Argentina, on pre‐exposure prophylaxis (PrEP) persistence, underscoring that persistence and retention are as important as initiation as PrEP programmes expand. Torres and colleagues [12] use objective pharmacologic measures to examine adherence, highlighting the limits of relying only on indirect adherence measures in a multicentre study carried out at sites in Brazil, Mexico and Perú. Together, these studies underscore that prevention services must respond to the lived realities of key populations rather than assuming that access alone is sufficient.

In the region, stigma remains one of the most persistent barriers to the HIV response. Balina and colleagues [13] evaluate a digital intervention among young adults in Peru, pointing to the potential of scalable communication strategies to reduce misinformation and create more enabling environments for testing, prevention and treatment. Sandoval and colleagues [14] show how HIV services can be integrated with sexual and reproductive health for female sex workers through intersectional collaboration. This contribution is especially relevant where gender, labour conditions, violence, legal vulnerability and health access intersect.

The issue also highlights how data infrastructure and regional research networks can support health‐system planning and accountability. Lyons and colleagues [15] show how data systems and patient‐centred care can support durable viral suppression in Trinidad and Tobago. Whereas Castilho and the CCASAnet cohort—Caribbean, Central and South America network for HIV Epidemiology— [16] document two decades of collaborative research across the region featuring sites from seven countries with over 61,000 adults and children living with HIV. This cohort collaboration demonstrates how sustained networks can build regional evidence, strengthen capacity, harmonize data and ensure that LAC populations are represented in the scientific literature.

Finally, Podchibiakin and colleagues [17] demonstrate the value of molecular epidemiology for understanding transmission dynamics and informing public health responses. Their study is a reminder that regional heterogeneity is not only social, economic and political; it is also virological.

LAC cannot be understood through a single narrative. The region is marked by inequities, but also by innovation, resilience and scientific leadership. The elimination of mother‐to‐child transmission of HIV and syphilis in Cuba in 2015 was followed by dual‐disease validation in several Caribbean territories and countries and more recent recognition of Brazil's progress towards elimination of vertical transmission of HIV, showing that ambitious public health goals are achievable when political commitment, primary care, antenatal testing, treatment access, laboratory systems and human‐rights‐based approaches are aligned [18]. These achievements also reflect the region's strong civil society and long history of rights‐based advocacy, coupled with strong academia and scientific ecosystems and, in several countries, robust public‐sector treatment programmes.

The articles included here do not cover every LAC territory, population or challenge. Together, however, they offer a powerful view of a region that is scientifically productive, politically complex, epidemiologically diverse and central to the future of the global HIV response. They also point out what is needed next: not a generic list of global priorities, but more equitable partnerships with and between LAC countries and communities. These partnerships should include fair access to innovation, meaningful regional participation in clinical trials and implementation studies, stronger regional manufacturing and procurement strategies, and sustained investment in community‐led services and social protection. In this context, the Alliance for the Elimination of HIV in the Americas, articulated by PAHO, offers a regional mechanism to strengthen coordination among countries, communities, technical agencies and other actors involved in the HIV response [19]. Rather than replacing the UNAIDS Global AIDS Strategy or WHO technical recommendations, the Alliance can help promote greater coherence, maintain the urgency required at this stage of the epidemic, and support countries in accelerating the adoption and implementation of priority interventions.

The central message of this issue is simple and urgent: the world needs to understand HIV in LAC, and LAC must be fully included in the global HIV agenda. This is the right moment to rethink how the region is represented, to rebuild partnerships on more equitable terms, and to rise to the challenges of ensuring that scientific breakthroughs translate into rapid, affordable and equitable delivery at scale. Ending AIDS as a public health threat will not be achieved if the needs and capacities of LAC remain peripheral to the global conversation.

## Author Contributions

**Claudia P. Cortes**: conceptualization, investigation, writing Original Draft, methodology, validation, writing Review Editing. **Carmen Pérez Casas**: conceptualization, investigation, writing OriginalDraft, methodology, validation, writing Review Editing. **Pedro Cahn**: conceptualization, investigation, writing Original Drafts, methodology, validation, writing Review Editing.

## Funding

This supplement was organized by IAS – the International AIDS Society – with financial support from Unitaid. The content of this supplement is solely the responsibility of the authors and does not necessarily represent the official views of the IAS or Unitaid.

## Conflicts of Interest

The authors declare no conflicts of interest.

## Data Availability

Data sharing not applicable to this article as no datasets were generated or analysed during the current study.
